# Harnessing the Potential of Natural Composites in Biomedical 3D Printing

**DOI:** 10.3390/ma17246045

**Published:** 2024-12-10

**Authors:** Farah Syazwani Shahar, Mohamed Thariq Hameed Sultan, Rafał Grzejda, Andrzej Łukaszewicz, Zbigniew Oksiuta, Renga Rao Krishnamoorthy

**Affiliations:** 1 Laboratory of Biocomposite Technology, Institute of Tropical Forestry and Forest Products (INTROP), Universiti Putra Malaysia, Serdang 43400, Selangor, Malaysia; farahsyazwani@upm.edu.my; 2 Department of Aerospace Engineering, Faculty of Engineering, Universiti Putra Malaysia, Serdang 43400, Selangor, Malaysia; 3 Prime Minister’s Department, MIGHT Partnership Hub, Aerospace Malaysia Innovation Centre (944751-A), Jalan Impact, Cyberjaya 63000, Selangor, Malaysia; 4 Faculty of Mechanical Engineering and Mechatronics, West Pomeranian University of Technology in Szczecin, 19 Piastow Ave., 70-310 Szczecin, Poland; 5 Institute of Mechanical Engineering, Faculty of Mechanical Engineering, Bialystok University of Technology, 45C Wiejska St., 15-351 Bialystok, Poland; a.lukaszewicz@pb.edu.pl; 6 Institute of Biomedical Engineering, Faculty of Mechanical Engineering, Bialystok University of Technology, 45C Wiejska St., 15-351 Bialystok, Poland; z.oksiuta@pb.edu.pl; 7 Smart Manufacturing Research Institute (SMRI), Universiti Teknologi MARA (UiTM), Shah Alam 40450, Selangor, Malaysia; rao@uitm.edu.my; 8 School of Civil Engineering, College of Engineering, Universiti Teknologi MARA (UiTM), Shah Alam 40450, Selangor, Malaysia

**Keywords:** natural composites, 3D printing, biomedical applications, additive manufacturing, sustainability

## Abstract

Natural composites are emerging as promising alternative materials for 3D printing in biomedical applications due to their biocompatibility, sustainability, and unique mechanical properties. The use of natural composites offers several advantages, including reduced environmental impact, enhanced biodegradability, and improved tissue compatibility. These materials can be processed into filaments or resins suitable for various 3D printing techniques, such as fused deposition modeling (FDM). Natural composites also exhibit inherent antibacterial properties, making them particularly suitable for applications in tissue engineering, drug delivery systems, and biomedical implants. This review explores the potential of utilizing natural composites in additive manufacturing for biomedical purposes, discussing the historical development of 3D printing techniques; the types of manufacturing methods; and the optimization of material compatibility, printability, and mechanical properties to fully realize the potential of using natural fibers in 3D printing for biomedical applications.

## 1. Introduction

Additive manufacturing (AM) is the process of creating three-dimensional objects from a digital file by building them layer by layer [1]. It is also known as 3D printing and is well known as a revolutionary technology that has transformed the method of manufacturing by changing the way products are designed, prototyped, and built. The manufacturing process involves adding material layer by layer to create a three-dimensional object, as opposed to traditional subtractive manufacturing methods that involve cutting, drilling, or milling away material to create a desired shape. AM has its roots in 1986, when Charles Hull developed the first 3D printing technology [2,3,4]. He invented a 3D printing process known as stereolithography (SLA), which uses a laser to solidify a liquid resin into a solid object. Since then, the technology has evolved significantly, with the emergence of new materials, processes, and applications emerging in mechanical engineering, with components joined by bolts [5,6,7].

One significant advantage of AM is the ability to create complex geometries and structures that are difficult or impossible with conventional methods [8,9]. This includes the ability to build hollow structures, lattice structures, and structures with varying densities, which can lead to significant weight savings and improved performance in certain applications. AM also enables greater design freedom and customization, as design changes can be made quickly and cost-effectively without the need for expensive tooling or molds [10]. Additionally, AM reduces lead times and increases efficiency by enabling rapid production of parts, often within hours or days [11]. AM has spread to various applications, such as aerospace, automotive, healthcare, and consumer products. In the aerospace industry, AM is used to create lightweight components and structures, reducing weight and improving fuel efficiency [12]. In health care, AM is used to create customized medical devices and implants, such as hearing aids, dental crowns, and hip replacements [13], allowing for a better fit and improved comfortability for patients. AM is also used to create medical models for surgical planning and training. Meanwhile, in the consumer products industry, AM is used to create customized products, such as jewelry and footwear [14]. It is also used in the production of prototypes and limited-edition products, allowing for faster times to market and lower costs.

Natural composites are increasingly attracting attention in biomedical applications due to a combination of environmental, regulatory, and technological drivers. Regulatory bodies such as the U.S. Food and Drug Administration and the European Medicines Agency are emphasizing the use of sustainable and biocompatible materials in medical devices, implants, and consumables to align with global health and safety standards [14,15]. These agencies are encouraging the development of biodegradable materials that reduce long-term environmental impact, particularly for single-use medical products. Furthermore, growing environmental concerns about plastic waste, coupled with international initiatives such as the European Green Deal [16] and the United Nations’ Sustainable Development Goals [17] have placed pressure on the healthcare sector to adopt greener materials. Biodegradable natural composites derived from renewable resources offer a promising solution by combining sustainability with the functional performance required for biomedical applications. In the healthcare industry, natural fibers are being increasingly explored for their biocompatibility, mechanical robustness, and ability to degrade harmlessly within the body or the environment. This shift not only addresses the rising demand for eco-friendly materials but also supports circular economy models in the medical sector, where waste reduction is a key priority.

The incorporation of natural fibers into composite materials marked a pivotal shift, driven by the need to enhance mechanical strength, reduce environmental impact, and better mimic the complex hierarchical structures of biological tissues. Early studies in the 1990s began integrating plant-based fibers into thermoplastic matrices for biomedical applications, but these efforts were hampered by challenges in compatibility and scalability [18]. In the 2010s, the maturation of 3D printing technologies like Fused Deposition Modeling (FDM) and SLA enabled more precise manipulation of natural composite materials, paving the way for applications in tissue engineering, prosthetics, and drug delivery systems [19]. Breakthroughs in nanocellulose- and lignin-derived composites further expanded the potential of natural fibers, offering nanoscale reinforcement that enhanced mechanical properties without compromising biodegradability.

The use of composites in AM offers versatility in reinforcement types, allowing for the use of chopped or continuous fibers like carbon fiber, fiberglass, and Kevlar, which can be integrated into the polymer material to create parts that are extremely lightweight yet as strong as metal [20,21]. The use of continuous fibers, in particular, enables the production of parts with exceptional strength and lightweight properties, expanding the possibilities for creating complex and high-performance components. Therefore, in the context of AM, composites can be used to enhance the properties of the final product. For example, metal composites can be used to improve the thermal properties of acrylonitrile butadiene styrene (ABS) polymers, which are commonly used in AM [22]. The rheological behavior of ABS/metal composites can be improved by optimizing the processing parameters, such as the temperature and speed of the AM process.

Research and development are ongoing in AM, with new materials, processes, and applications being explored. This includes the development of new materials, such as biodegradable plastics and conductive materials, as well as new processes, such as multi-material printing and hybrid manufacturing. The future of AM is promising, with the potential to transform industries and society. Thus, this review aims to discuss AM technologies and materials development, as well as the possibilities of using 3D-printed composites as alternative materials in biomedical applications.

## 2. Historical Development of Additive Manufacturing

AM history can be traced back to 1945, when Murray Leinster introduced the concept as a part of a short science fiction story. The general concept and procedure were briefly described in the story as “feeding plastics into a moving arm as it draws in the air following the scanned drawings. Plastics came out of the drawing arm and hardened as a 3D model structure as it follows the drawings” [23]. Leinster’s fiction later became a true science breakthrough in 1971, as Johannes F Gottwald filed his theory as a patent titled “Liquid metal recorder” with patent ID US3596285A [24]. The patent described the process of inkjet printing, whereby a metal is heated until the melting point and forced through a moving nozzle. The material then solidifies and forms a pre-defined structure. However, the invention of the technology was not achieved until the patent’s expiration date was reached. In 1974, David E. H. Jones, under his pseudonym, “Daedalus”, revealed the possibility of the 3D printing concept. His musings were covered in the New Scientist magazine under a regular segment titled “Ariadne” [25]. Jones perfectly described the process of SLA, which would become one of the fundamental principles for an invention that would be developed a decade later. He described a column containing a laser beam with higher-energy particles than ultraviolet light leaving a track of polymers behind. These polymers solidify and create a 3D model structure of a predefined object. However, Jones only presented this idea as a joke and a part of his musings on future technologies; thus, he was never credited with this idea.

**Figure 1:**
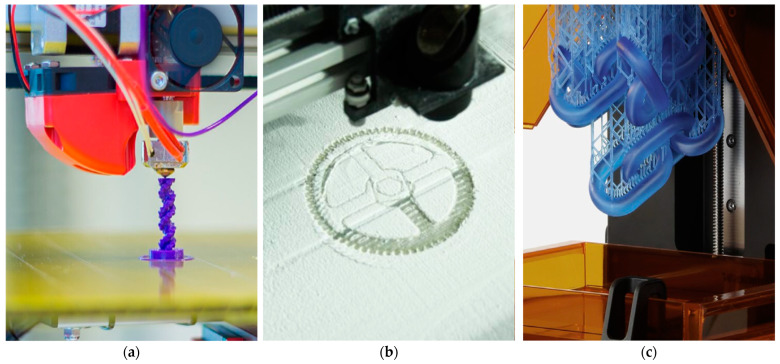
Main AM technologies: (**a**) FDM (Source: Jonathan Juursema, CC BY-SA 3.0 via Wikimedia Commons) [31]; (**b**) SLS [32]; (**c**) SLA (Source: https://formlabs.com/3d-printers/ (accessed on 5 December 2024), CC BY 2.0 via flickr) [33].

In the 1980s, AM became a reality. According to Matthew A. McIntosh, Hideo Kodama was the first Japanese inventor to file a patent describing the laser-beam curing process, also known as rapid prototyping. The idea was titled “Stereoscopic figure drawing device” with patent ID JPS56144478A [23,26]. However, due to a restricted budget, the project was abandoned a year later. Following that, in 1982, Raytheon Technologies Corp patented the “Method for fabricating articles by sequential layer deposition” with patent ID US4323756A [27]. This patent describes the deposition of multiple thin layers of particles using a laser beam and the fusion of each layer to form a complete 3D structure. In 1984, stereolithography was, once more, patented by French inventors with patent ID FR2567668A1 [28]. In this patent, a complete stereolithography process was described in detail. However, similar to Dr Kodama’s situation, the project was terminated due to limited market potential. Since then, many ideas on AM were patented. However, due to the lack of funding, most projects were abandoned. Three weeks after the French inventors filed their patents, Charles Hull also filed his patent on SLA fabrication with patent ID US4575330A [2]. His patent was approved in 1986, and he started his own company known as 3D Systems. Two years later, the first commercial SLA 3D printer known as SLA-1 was released. Not far behind the commercialization of SLA, FDM and Selective Laser Sintering (SLS) followed closely. In 1987, the first SLS machine, nicknamed Betsy, was invented by Texas University student Carl Deckard and his advisor, Dr. Joe Beaman [29]. SLS works by hardening and fusing small particles of metals, plastics, glass, or ceramics using a laser, unlike SLA, which uses the laser to cure the resin into a pre-determined shape. Although the basic working principle is the same, the materials used in the two methods are completely different. Two years later, Deckard and Paul Forderhase created a second-generation SLS machine nicknamed Bambi. Within the same year (1989), S. Scott Crump, a co-founder of Stratasys, filed a patent for FDM with patent ID US5121329A [30]. The patent was approved in 1992, and the first commercialized FDM printer was released in the same year. With the three main patented technologies for 3D printing began the rise of 3D printer manufacturers and CAD tools. Moreover, to date, FDM has become the most used technology in 3D printers. Figure 1 shows the three main AM technologies (FDM, SLS, and SLA) developed before the innovation of new AM technologies.

The 1990s saw the development of other AM technologies and CAD tools. Technologies were further improved, and more CAD tools were introduced. EOS GmbH was founded by Dr. Hans J. Langer and Dr. Hans Steinbichler, providing high-end rapid prototyping [34]. In 1990, BMW was the first customer to buy the EOS “Stereos” system, and its technology has been recognized worldwide ever since. Furthermore, with FDM patented in 1992, many FDM printers were developed for both industry and individuals [30]. In 1994, Solidscape was founded, a company that produces 3D printers exclusively for jewelers [35]. The printer offers higher-resolution 3D printing abilities to create the most intricate jewelry with high accuracy and precision. Z Corporation (later acquired by 3D Systems Corporation) also acquired proprietary rights from MIT and subsequently engineered the Z402, a 3D printer utilizing inkjet printing technology [36,37].

The 2000s saw significant advancements in the materials and processes used in AM, and 3D printing started to gain media visibility. This was because the patents on all three main 3D printing technologies were set to expire, making them freely open to the public masses for experimentation and improvements [2,30,38]. New materials, such as conductive filaments and biocompatible materials, were developed, expanding the applications of the technology. Processes were also improved, with the development of Multi-Jet Modeling (MJM) [39] and Direct Metal Laser Sintering (DMLS) [40]. Furthermore, with the emergence of new printing materials, research in the medical field also began to rise. The first 3D-printed functional experimental solid organ, a miniature kidney that could secrete urine, was created in 2003 by researchers from Wake Forest University [41]. With the kidney as the precedent, tissue engineering started to expand with the use of 3D printing. Although these 3D-printed tissues and organs were still not ready to be transplanted into a human body, the growth of experimentation in this area had increased rapidly and gained considerable traction over the years. 2005 marked the start of the wide distribution of 3D-printed parts and DIY 3D printers due to the open-source movement (RepRap movement) initiated by Bowyer [42,43]. The idea of the open-source movement is to enable the creation of a self-replicating printer, which reduces the component costs, making them more accessible and easier to distribute. Thingiverse was the first website launched in 2008 to gather 3D printing enthusiasts to share 3D printing models with the public [44]. In the same year, the first 3D-printed prosthetic was patented by Summit [45]. Furthermore, with the addition of 3D scanning, the manufacturing of prostheses and orthoses became much faster and cheaper, with parts optimized to perfectly fit patients. Finally, 2009 marked the end of the three main 3D printing technologies with FDM patents going into the public domain. Thus, the era of 3D printing innovation started making 3D printers cheaper and more accessible, in addition to increasing visibility among the masses.

The 2010s marked the rise of industrial AM, with the technology being used for the production of end-use parts. Companies such as GE Aviation began using AM for the production of components for aircraft engines and power generation turbines [46]. In 2014, the first fully 3D-printed car (Strati) was developed and showcased at the International Manufacturing Technology Show in Chicago [47,48]. In addition, new 3D printing materials were explored and developed. Printing materials were becoming stronger and cheaper. Each new material was being developed to satisfy the current needs of the market. Some examples are heat-resistant resins [49], flexible plastics [50], and strong carbon fiber filaments [51], among many more. In 2016, Dr Daniel Kelly’s lab managed to create a 3D-printed bone tissue using bio-ink [52,53,54]. In 2020, PPE uniforms were needed to combat COVID-19; thus, 3D printing was used to manufacture these uniforms rapidly in large quantities within a short time frame to ensure that health workers and front liners were well-equipped and protected [55].

**Figure A1:**
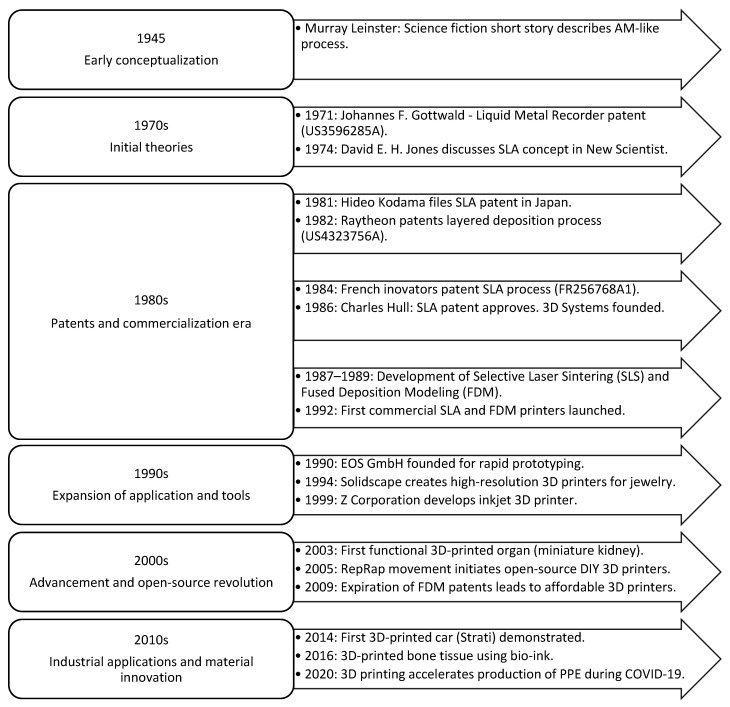
Technological progression of additive manufacturing.

The future of AM is promising, with advancements in technology and materials continuing to expand the applications of the technology. The development of 3D printing is expected to revolutionize the industry. Furthermore, armed with the use of artificial intelligence and machine learning, it is also expected that there will be improvements in the efficiency and accuracy of AM processes. Figure A1 (located in Appendix A) shows the timeline of the development of additive manufacturing technology.

## 3. Types of Additive Manufacturing

All 3D printing processes share a commonality in their additive nature, where objects are built layer by layer. This AM approach distinguishes 3D printing from traditional subtractive manufacturing methods. Each 3D printing process, whether it is SLA, SLS, FDM, Digital Light Processing (DLP), Multi Jet Fusion (MJF), PolyJet, DMLS, or Electron Beam Melting (EBM), involves adding material layer upon layer to create a three-dimensional object. This layer-by-layer construction allows for intricate designs, complex geometries, and customization that traditional manufacturing techniques may struggle to achieve. Additionally, 3D printing offers greater design freedom, enabling the creation of single-piece parts and reducing the need for assemblies, which is a distinctive feature across all 3D printing technologies. There are seven main categories of 3D printing technologies, which are discussed in this section.

### 3.1. Material Extrusion

**Figure 2:**
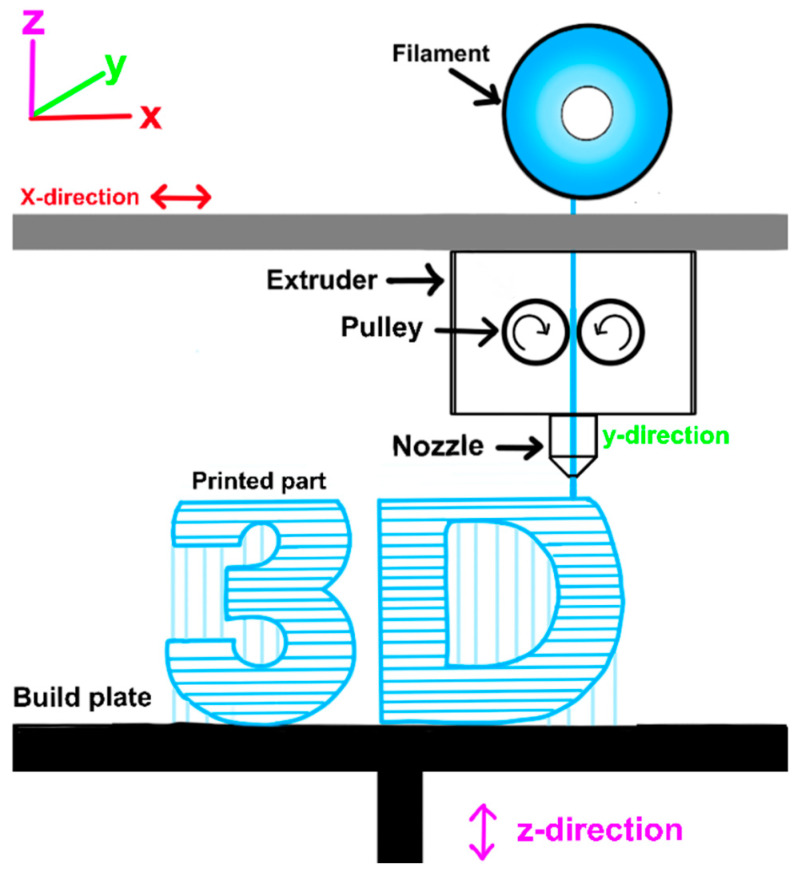
MEX process.

Material extrusion (MEX) is a fundamental 3D printing technique that involves the deposition of material through a heated nozzle, which then solidifies to form layers. This process allows for the creation of intricate geometries that would be challenging to achieve using traditional manufacturing methods. In material extrusion, a material, usually in a semi-molten, softened, or viscous state, is forced through a nozzle under controlled pressure or temperature conditions, then deposited layer by layer along pre-determined paths to fabricate a three-dimensional object, with each layer solidifying or adhering upon cooling or curing, as shown in Figure 2. The additive nature of this process enables the creation of complex shapes with high precision. Some examples of technologies categorized as MEX are FDM and Direct Ink Writing (DIW).

One of the most notable advantages of MEX is its scalability for size [56]. Unlike resin-based technologies such as SLA or powder-based methods like SLS, extrusion systems can be scaled to produce significantly larger parts at a fraction of the cost. This capability makes MEX particularly suited for industries requiring large-scale prototyping or the creation of full-sized functional components without incurring prohibitive expenses. The relatively simple mechanics of extrusion systems contribute to their ability to maintain performance and reliability at larger scales, a feat less feasible with more complex systems reliant on lasers or photopolymerization.

Ease of maintenance and operation is another distinguishing feature of MEX technology [57]. The mechanical simplicity inherent in these systems makes them more accessible to non-expert users and reduces the cost and complexity of repairs. In contrast, resin-based systems, which rely on UV-sensitive screens and precise optical components, or powder-based technologies with intricate laser mechanisms typically require more specialized maintenance. The durable nature of extrusion hardware further contributes to its reliability and longevity, enhancing its appeal for long-term use in both professional and personal settings.

MEX also excels in multi-material and multi-color capabilities [58]. The availability of dual extruder systems enables users to print with multiple materials or colors in a single process, which is not as straightforward in other technologies. This feature is particularly valuable for creating parts with complex geometries, such as those requiring rigid structures combined with flexible components, or for aesthetic applications involving multiple colors. The ability to use soluble support materials in conjunction with primary filaments further simplifies the production of intricate designs, expanding the range of possibilities in design and manufacturing.

Finally, the operational simplicity of MEX is a significant advantage [57]. Unlike other 3D printing technologies that often require tightly controlled environments—such as UV-sensitive setups for resin-based systems or inert gas chambers for metal powder-based methods—MEX operates effectively under standard room conditions. This flexibility not only reduces operational costs but also simplifies the integration of extrusion-based printers into diverse environments, including homes, offices, and educational institutions.

Despite its benefits, MEX faces challenges such as surface finish quality and dimensional accuracy [59]. Furthermore, even though MEX offers versatility and cost-effectiveness, it is inherently prone to anisotropy in the mechanical properties of the printed components [60]. This anisotropy arises due to the layer-by-layer deposition process, where the strength of the material often varies significantly between the layers (z direction) and within the layers (X–Y plane). Such disparities can lead to reduced performance in load-bearing or dynamically stressed applications. Addressing this challenge requires the optimization of process parameters such as nozzle temperature, layer adhesion, and printing speed, as well as the exploration of advanced composite materials that promote isotropic properties. Future advancements in material science and extrusion techniques may mitigate these issues and enhance the uniformity of mechanical properties across all dimensions.

Researchers and industry experts are continuously working to address these challenges to enhance the capabilities of material extrusion. The future of MEX in 3D printing looks promising, with ongoing research focusing on improving material properties, print speeds, and scalability. Advancements in materials science and technology are expected to further expand the applications of MEX in diverse fields.

MEX is a pivotal technique in 3D printing that has transformed the manufacturing landscape. With its versatility, precision, and cost-effectiveness, MEX continues to drive innovation across industries, paving the way for a future where complex designs can be realized with ease and efficiency.

### 3.2. VAT Photopolymerization

**Figure 3:**
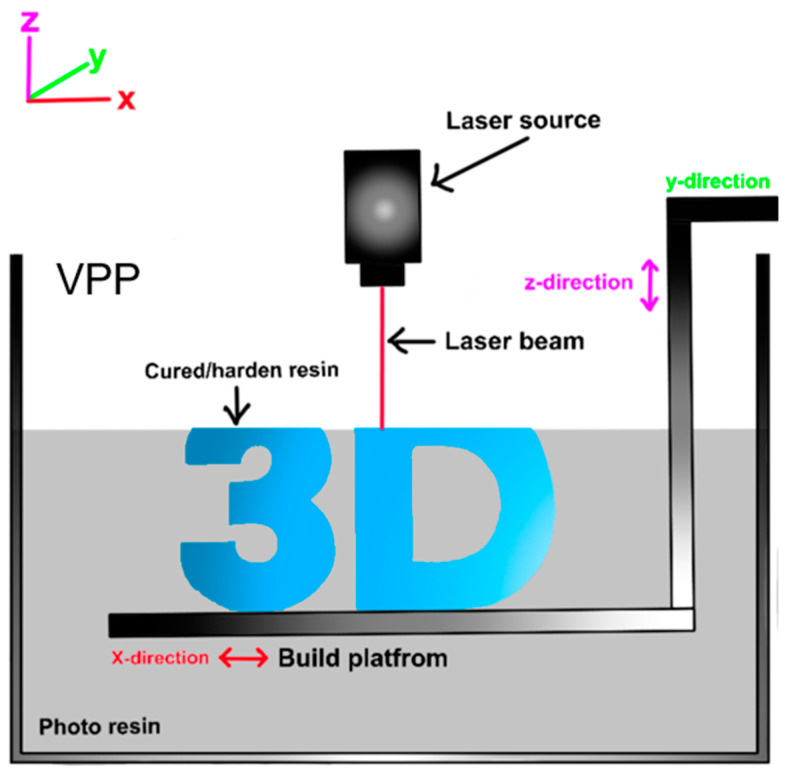
VPP process.

VAT Photopolymerization (VPP) is a type of AM technology that produces 3D objects by selectively curing a photopolymer liquid resin using light waves [61]. This process is commonly used in various 3D printing applications due to its ability to create highly detailed and precise parts. The basic principle of VPP involves a container filled with a liquid photopolymer resin. A light source, such as a laser or digital light projector, is used to selectively cure the resin layer by layer, building up the 3D object. In VPP, the curing mechanism involves a chemical process known as crosslinking, where the photopolymer resin undergoes polymerization upon exposure to specific wavelengths of light. During this process, the resin’s molecules form covalent bonds, creating a tightly interconnected network or “cross-linked” structure. This cross-linking solidifies the liquid resin layer by layer, resulting in a robust and stable 3D object. As each layer is cured, the build platform is lowered, allowing a new layer of resin to flow beneath the object, and the process is repeated until the entire part is fabricated. The extent of crosslinking directly influences the mechanical properties, durability, and thermal resistance of the final product, making it a critical factor in determining the performance of VPP components. Figure 3 shows the basic setup of the VPP process.

There are several types of VPP technologies, including SLA, digital light processing (DLP), and Continuous Digital Light Processing (CDLP). SLA uses a laser to trace the cross-sectional area of each layer, while DLP and CDLP employ a digital light projector to cure an entire layer at once, potentially resulting in faster print speeds. The materials used in VPP are known as photopolymers, which are polymers that undergo a chemical change when exposed to specific wavelengths of light, causing their molecules to link together and solidify. These materials can be formulated to have a wide range of properties, such as standard resins, tough and durable resins, elastic and flexible resins, and even biocompatible resins for medical applications.

One of the key advantages of VPP is its ability to produce parts with high degrees of detail, precision, and overall quality. This makes it well-suited for applications such as jewelry, low-run injection molding prototypes, and various dental and medical applications. However, VPP also has some limitations. The produced parts can be relatively brittle and may require post-curing to achieve significant strength. Additionally, the material choices are often limited, and the process can be more costly compared to other AM technologies. Despite these challenges, VPP continues to be a popular and widely used AM technique, particularly in industries where high-quality, detailed parts are required.

As technology continues to evolve, researchers are exploring ways to address limitations and expand the range of materials and applications. In recent years, there has been a growing interest in the use of functional dyes in polymeric 3D printing, including VPP [62]. These dyes can be incorporated into photopolymer resins to impart additional properties, such as color, luminescence, or even stimuli-responsive behavior. This opens up new possibilities for the design and fabrication of advanced 3D-printed materials and devices. As the field of AM continues to advance, VPP is likely to play an increasingly important role in the development of innovative products and applications across various industries. Ongoing research and technological advancements will continue to expand the capabilities and versatility of this AM technique.

VPP is a powerful AM technology that enables the creation of highly detailed and precise 3D objects through the selective curing of photopolymer resins. While it has some limitations, its unique capabilities make it a valuable tool in a wide range of applications, and its continued evolution promises even more exciting developments in the future.

### 3.3. Powder Bed Fusion

**Figure 4:**
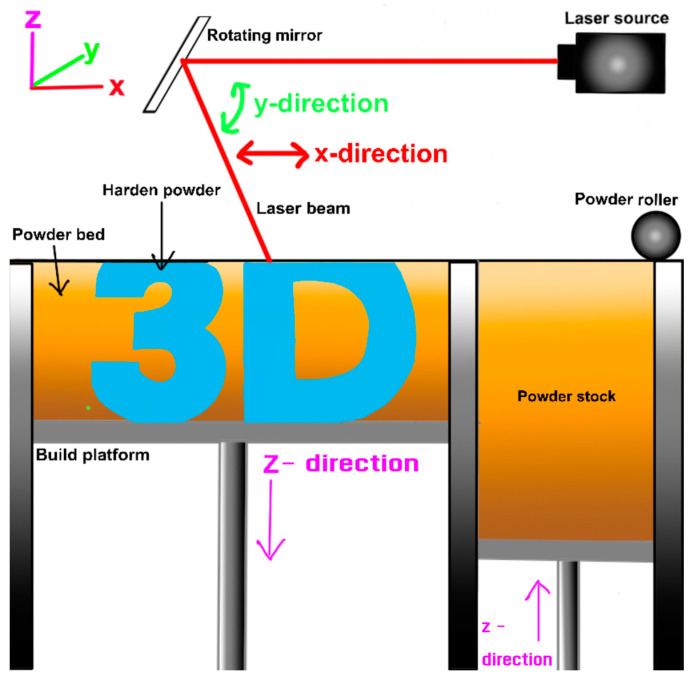
PBF process.

Powder Bed Fusion (PBF) is a widely used AM process that uses a high-energy heat source, such as a laser or electron beam, to selectively fuse layers of powdered material within a bed to create a three-dimensional object [63]. PBF is one of the most common powder bed fusion techniques. In this process, a thin layer of powdered material is spread across a build platform using a re-coater or roller, as shown in Figure 4. A high-energy laser beam then selectively melts and fuses the powder particles in the desired areas based on a digital 3D model. After each layer is fused, the build platform lowers, and a new layer of powder is spread on top. This process is repeated layer-by-layer until the entire 3D part is built. The unfused powder around the part acts as support, allowing for the creation of complex geometries that would be difficult or impossible to produce using traditional manufacturing methods.

The powder used in PBF is typically very fine, with particle sizes ranging from 15 to 45 μm [62]. This fine powder allows for the creation of parts with high resolution and excellent surface finish. However, the small particle size also means the powder can be a respiratory and flammability hazard, requiring proper safety equipment and handling procedures. The laser used in PBF-LB and the electron beam used in PBF-EB are precisely controlled by galvanometer mirrors, allowing them to move quickly and accurately across the powder bed to selectively melt the desired areas. The laser power, scan speed, and other parameters are carefully optimized to ensure proper fusion of the powder and the creation of a high-quality part. After the part is built, the remaining unmelted powder is removed and can be recycled for future use, reducing material waste. The part may then undergo additional post-processing steps, such as heat treatment or surface finishing, to improve its mechanical properties or appearance.

PBF is used to produce a wide range of parts, including medical implants, aerospace components, automotive parts, and industrial tools. The process allows for the creation of complex geometries, internal features, and customized designs that would be difficult or impossible to achieve using traditional manufacturing methods. However, PBF is not without its challenges. The process can be sensitive to various parameters, such as powder quality, laser power, and environmental conditions, which can affect the quality and consistency of the final part. Careful process optimization and monitoring are essential to ensure successful PBF production. Some examples of PBF technology are SLS (PBF-LB), DMLS (PBF-LB), EBM (PBF-EB), and MJF.

Overall, powder bed fusion—specifically LPBF—is a powerful AM technology that enables the creation of highly complex and customized metal parts. Its ability to produce parts with intricate geometries and high precision has made it an increasingly important tool in various industries, from aerospace to medicine. In summary, powder bed fusion—particularly laser powder bed fusion—works by selectively melting and fusing layers of fine metal powder to build up a 3D part. This process allows for the creation of complex geometries and customized designs, making it a valuable tool in modern manufacturing.

### 3.4. Material Jetting

**Figure 5:**
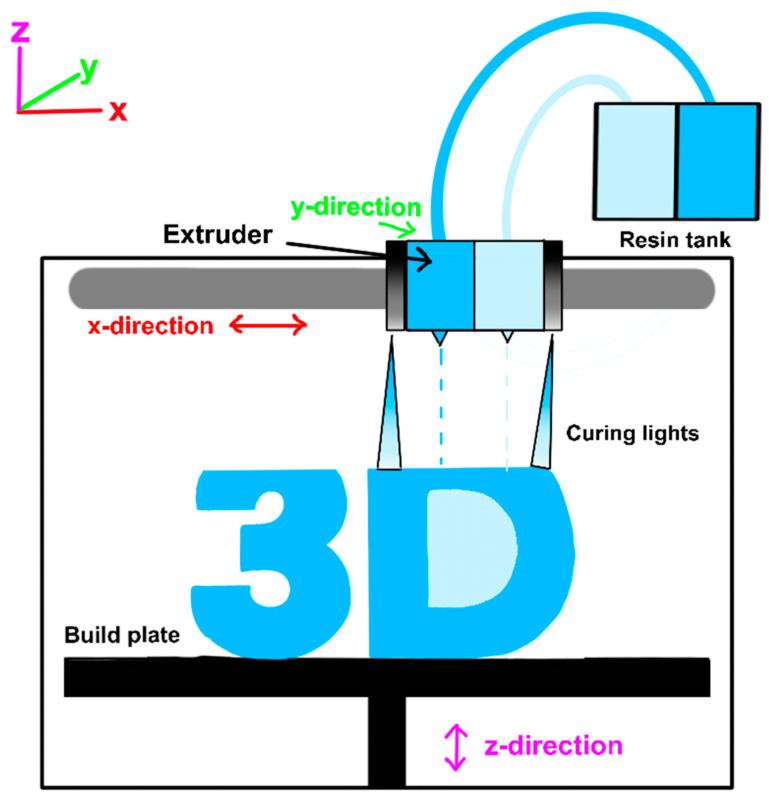
MJT process.

Material Jetting (MJT) is a 3D printing technology that works similarly to inkjet document printing, but instead of jetting drops of ink onto paper, it jets drops of liquid photopolymer onto a build tray [64]. Multiple print heads jet the material simultaneously to create each layer, and UV light is then used to cure the layers. These layers build up one at a time in an additive process to create a 3D model, as shown in Figure 5. Some examples of MJT printing technologies are Poly-Jet Modeling (PJM) and Multi-Jet Modeling (MJM).

One of the key advantages of MJT is its ability to combine different print materials within the same 3D-printed model in the same print job [64]. This allows for the construction of functional assemblies, reducing the need for multiple builds. The process can create parts with a matte or glossy surface finish and can print using a variety of rigid and flexible materials, as well as gradient combinations of materials. MJT is known for its high print speed and accuracy, making it well-suited for producing multi-color, multi-material parts [65]. The technology has an X–Y resolution ranging between 600 DPI and 1600 DPI (depending on the printer dimensions) and can print layers as thin as 16 μm or 30 μm [66]. This level of precision allows for the fabrication of intricate, detailed parts. In addition to the primary model material, material jetting also utilizes a gel-like support material to facilitate the successful printing of complicated geometries. This support material can be removed by hand or by a high-powered water jet station after the part is fully cured.

The materials used in MJT are typically photopolymers, which are liquid resins that solidify when exposed to UV light [65]. These materials can be formulated to have a wide range of mechanical [67], thermal [68], and optical properties [69], allowing for the creation of parts with diverse functional capabilities. Researchers have also explored the use of composites (matrices in the form of liquid suspensions, slurries, or photocurable resins) in MJT, incorporating fillers such as ceramics [70], metallic inks [71], or composite resins [67] to enhance the properties of the printed parts. This can lead to improvements in areas like strength [72], stiffness [67], or electrical conductivity [73] depending on the specific composite formulation. The process parameters in MJT, such as tray location and build orientation, can have a significant impact on the final part quality and properties [65]. Extensive research has been conducted to understand the effects of these parameters and optimize the process for different applications.

One of the key challenges in MJT is ensuring the dimensional and geometric precision of the printed parts, particularly for micro-scale features [74]. Researchers have developed metrological benchmarks and assessment methods to evaluate the accuracy and reproducibility of MJT processes. Despite these challenges, MJT has found widespread applications in various industries, including rapid prototyping, product design, medical devices, and even the production of functional end-use parts. The technology’s ability to combine multiple materials and produce high-quality, detailed parts has made it a valuable tool in the AM landscape.

As the field of MJT continues to evolve, researchers and manufacturers are exploring new materials, process optimizations, and applications to further expand the capabilities of this versatile 3D printing technology.

### 3.5. Binder Jetting

**Figure 6:**
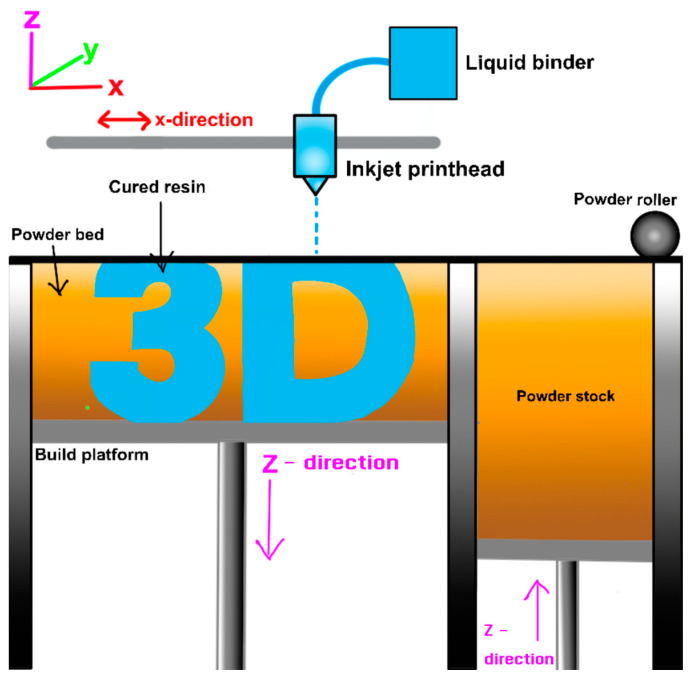
BJT process.

Binder Jetting (BJT) is a type of AM process that uses a powder-based material and a liquid binder to create 3D objects [75]. This process is considered one of the fastest AM techniques and is well-suited for mass production of small, accurate parts. The BJT process can be broken down into five main steps, which start with powder deposition, followed by binder deposition, layer lowering, and post-processing. In the BJT process, a thin layer of powder material, such as metal [76], ceramic [77], or polymer [78], is spread over the build platform using a roller or re-coater mechanism. Then, a print head selectively deposits a liquid binder onto the powder layer, binding the particles together in the desired pattern. The build platform is then lowered by the thickness of the next layer set in the printing setup. The process of powder deposition, binder deposition, and layer lowering is repeated until the entire 3D object is formed as shown in Figure 6. After the printing process is complete, the part may undergo additional post-processing steps, such as curing [79], sintering [80], or infiltration [81], to improve its mechanical properties and dimensional accuracy. An example of 3D printing technology classified under BJT is Layerwise Slurry Deposition (LSD).

One of the key advantages of BJT is its ability to use a wide range of materials, including metals, ceramics, and polymers [82]. This versatility allows for the production of parts with diverse mechanical and physical properties, making it suitable for a variety of applications. Another advantage of BJT is its speed and cost-effectiveness [83]. The process is generally faster than other AM techniques, as it does not require the use of a laser or other energy-intensive equipment. Additionally, the lack of support structures required in BJT can further reduce the time and cost of part production.

However, BJT also has some limitations. The parts produced by this process can have worse mechanical properties compared to those made by other AM techniques, such as SLM or FDM [84]. This is due to the inherent porosity of the parts, which can be reduced by post-processing steps like sintering or infiltration. Another challenge with BJT is the accuracy and dimensional stability of the final parts [85]. The shrinkage and warping that can occur during post-processing can make it difficult to predict the final dimensions of the part. Careful design and compensation for these effects are necessary to ensure the desired part geometry.

Despite these challenges, BJT has found applications in a wide range of industries, including the aerospace, automotive, and medical fields. In aerospace, this technology is used for the production of complex, lightweight parts and components [86], whereas automotive applications focus on rapid prototyping and the production of customized parts [87]. BJT is also used in the medical sector to fabricate patient-specific implants and prosthetics [88]. The ability to produce complex, customized parts quickly and cost-effectively has made BJT a valuable tool in the AM landscape.

### 3.6. Directed Energy Deposition

Directed Energy Deposition (DED) stands as a prominent category within AM, leveraging metallic materials in either powder or wire form. This technology is currently experiencing significant growth and research interest, representing a promising AM method. In DED, products are formed by melting a baseplate using a laser beam and subsequently depositing powdered material into the resulting melt pool [89]. Through precise control of laser power and material deposition, DED achieves the desired shape of metal parts.

**Figure 7:**
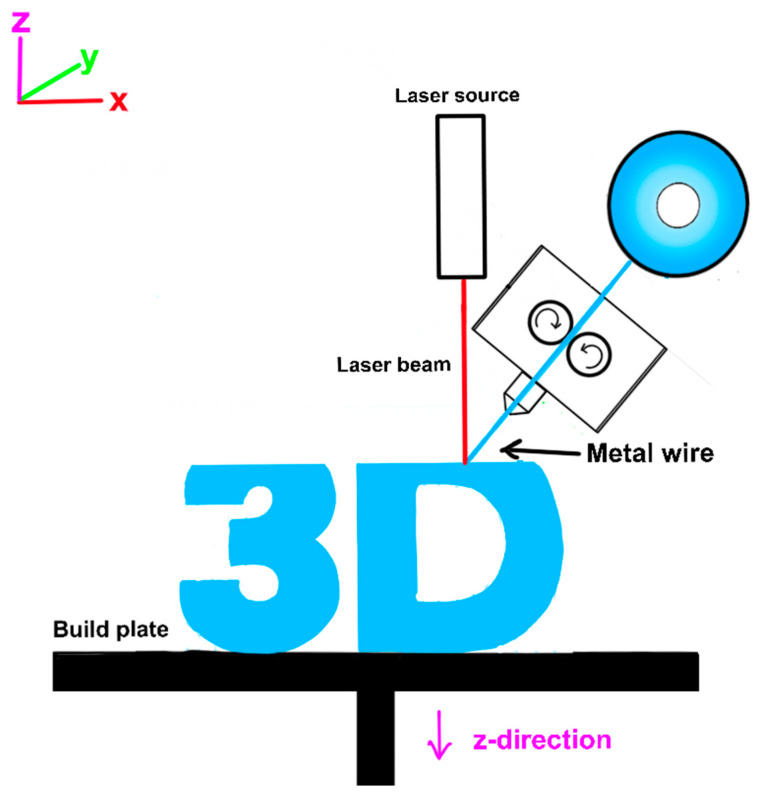
DED process.

All iterations of DED incorporate a Powder Delivery System (PDS), typically comprising a single or multiple coaxial nozzles with localized shielding of the melt pool and feed lines for powdered material. Powder may be introduced via an inert carrier gas or gravity feed, alongside a separate supply of shielding gas to safeguard the molten weld pool from oxidation. DED plays a crucial role in creating near-net-shaped components, streamlining production by reducing processing steps such as cutting and finishing while also conserving raw materials. This versatility makes DED ideal for tasks like coating, repairing, and constructing parts with highly intricate geometries. Figure 7 shows the DED process.

One of the key benefits of DED is the high degree of control over the grain structure, which makes it particularly suitable for repair work on high-quality, functional parts [90]. The process also allows for the ability to feed different powders simultaneously into the melt pool without limiting part dimensions [91]. DED offers several other advantages, including minimal distortion of the workpiece [92], reduced heat-affected zones [93], and better surface finishes compared to other AM processes. However, a balance is needed between surface quality and speed, and in some cases, speed can be sacrificed for higher accuracy and a pre-determined microstructure, especially in repair applications.

The DED process uses either wire or powder as the feedstock material [94]. The use of wire is less accurate due to the pre-formed shape, but it is more material-efficient compared to powder, as only the required material is used. The method of material melting varies between laser, electron beam, and plasma arc melting, all within a controlled chamber with reduced oxygen levels. The movement of the feed head in DED machines can be either with a fixed, vertical deposition or with four- or five-axis machines, where the feed head can move around a fixed object. The choice of approach depends on the specific application and the object being printed. The cooling time of the deposited material in DED is very fast, typically between 1000 and 5000 degrees Celsius per second, which affects the final grain structure of the deposited material. The overlapping of the deposited layers can also cause re-melting, resulting in a uniform but alternating microstructure. Typical layer thicknesses in DED range from 0.25 mm to 0.5 mm, and the print speed can vary depending on the specific machine and application, with some machines capable of depositing two layers per minute. Some variants of DED process are Direct Metal Deposition (DMD) and Direct Metal Tooling (DMT).

Despite the advantages of DED, there are also some limitations. The finishes can vary depending on the material used, and post-processing may be required to achieve the desired effect [95]. Additionally, the limited material use and the need for further research to advance the fusion processes to a more mainstream positioning are some of the disadvantages of DED technology [96].

DED is a rapidly advancing field, with ongoing research and development aimed at addressing the current limitations and expanding the capabilities of the technology. Researchers are exploring ways to improve the surface finish [97] and further enhance control over the microstructure of deposited materials [98]. One area of active research in DED is the development of multi-material deposition systems, which would allow for the simultaneous use of different metal powders or the integration of non-metallic materials such as ceramics or polymers into the deposition process [99]. This could lead to the creation of novel, functionally graded materials with tailored properties for specific applications. Another focus of research in DED is the optimization of the process parameters, such as the laser power, scan speed, and powder feed rate, to improve the overall efficiency, productivity, and part quality [100]. Advancements in sensor technology and real-time process monitoring are also being explored to enable better control and monitoring of the DED process [101].

As DED technology continues to evolve, it is expected to find increasing applications in industries where the ability to repair, rebuild, and customize high-performance parts is of critical importance. Ongoing research and development efforts in this field are aimed at addressing current limitations and unlocking the full potential of DED as a transformative AM technology.

### 3.7. Sheet Lamination

**Figure 8:**
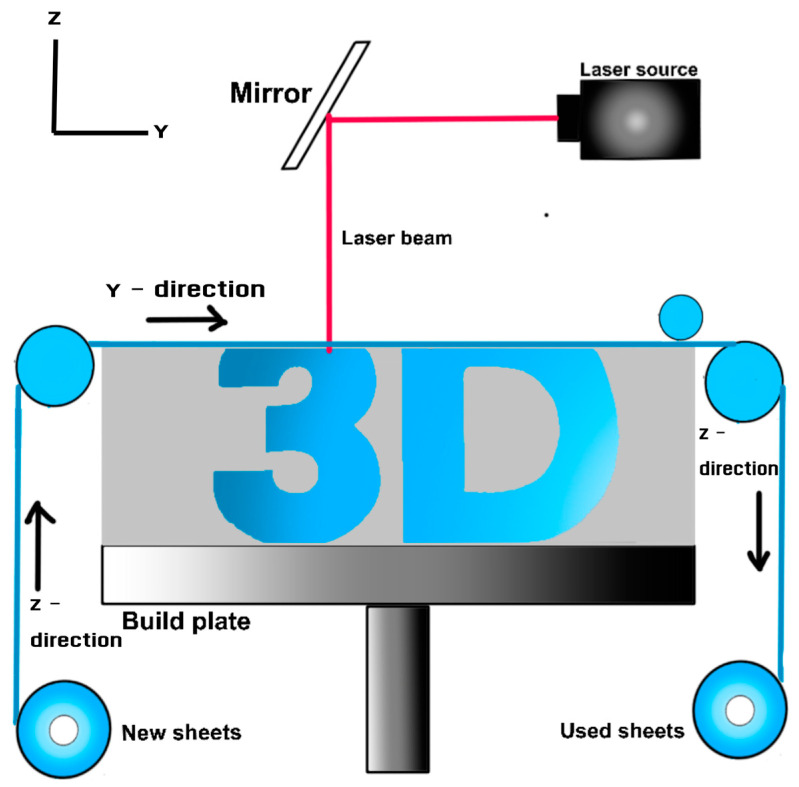
SHL process.

Sheet Lamination (SHL) is a 3D printing technology that has shown untapped potential in terms of speed and versatility. This method involves using sheets of materials like paper, plastic, or metal, which are laminated together to create 3D objects [102]. Unlike other 3D printing technologies, SHL utilizes whole layers of building material, either as individual sheets or material on a roll. The process involves rolling the material over the construction area, gluing it if necessary, and then cutting the part free along the way, resulting in the creation of the desired object, as shown in Figure 8.

Traditionally, SHL has been used for architectural landscape models, where can be added to the paper [103]. However, prints made using this method lack mechanical strength and are primarily for display purposes. Despite limitations in material options, such as being restricted to plastic, metal, or paper, SHL offers a wide range of applications, from architectural parts to functional components. The technology’s versatility allows for the creation of various objects, depending on the material used and the desired outcome. Some examples of SHL technologies are Laminated Object Manufacturing (LOM) and Metal Laminated Tooling (MELATO).

One of the key advantages of SHL is its potential to optimize the speed of 3D printing processes. Speed is a crucial factor in 3D printing, as faster printing can lead to increased production and reduced costs. SHL offers a fast printing process, which is essential for industrial applications where speed and efficiency are paramount [104]. Moreover, manufacturing with SHL occurs at low temperatures, eliminating the need for high melting points and reducing energy consumption [105].

The use of SHL in industrial settings is still not widespread, but its potential for speed and efficiency makes it a promising technology for various industries. By leveraging the rotational process of SHL, companies can enhance their production capabilities and streamline their manufacturing processes. The technology’s ability to produce parts quickly and at low temperatures makes it suitable for industrial applications where speed and cost-effectiveness are critical factors.

In conclusion, SHL is a 3D printing technology with significant untapped potential, particularly in terms of speed and efficiency. By utilizing sheets of materials like paper, plastic, or metal, this method offers a unique approach to creating 3D objects. Despite its historical use in architectural models, SHL has evolved to cater to a broader range of applications, from functional parts to industrial components. The technology’s speed, low-temperature manufacturing process, and potential for optimization make it a promising option for industries looking to enhance their production processes and reduce costs.

## 4. Natural Fiber Composites as an Alternative Materials for 3D Printing

### 4.1. Introduction to Natural Fibers for 3D Printing

Natural fibers represent a promising frontier in the realm of sustainable materials for 3D printing applications. These fibers, derived from renewable sources, not only offer an eco-friendly alternative to synthetic reinforcements but also enhance the mechanical properties of 3D-printed components [106,107,108]. However, their adoption in additive manufacturing is not without challenges, necessitating an organized exploration of their characteristics, advantages, and limitations. Current trends are mostly focused on FDM due to the ability to customize material for the melt extrusion process. Other than FDM, some research has also been conducted on SLA, although such works are still in preliminary stages. Table 1 shows previous research on 3D printing using natural fiber composites.

While there have been some efforts to develop 3D printing technologies specifically designed for natural fibers, such as pellet extrusion or SLA, these approaches are still relatively new and face their own set of challenges. However, as technology advances, there are some possibilities of seeing improvements in the ability to use natural fibers in a wider range of 3D printing applications.

### 4.2. Characteristics, Challenges, and Compatibility of Natural Fibers for 3D Printing

Natural fiber-reinforced composites (NFRCs) have gained attention in 3D printing due to their eco-friendly and cost-effective properties. When natural fibers are added to thermoplastics like ABS and PLA, the mechanical properties of the 3D-printed objects are affected. There have been several studies investigating the properties of 3D-printed natural fiber composites under different conditions. Table 2 shows the tensile properties of the 3D-printed natural fiber composites that were tested based on ASTM D638 [117].

The integration of natural fibers into 3D printing presents several opportunities for improving material sustainability and performance. However, challenges such as moisture sensitivity [118], inconsistent fiber morphology [119], and poor interfacial adhesion [120] between fibers and polymers persist. For instance, while fibers like kenaf [56,107] and hemp [111] demonstrate strong mechanical reinforcement potential, their moisture absorption can lead to warping and reduced print quality. Wood dust, though easy to process, offers limited mechanical advantages [110], while agave fiber’s brittleness at higher loadings restrict its application range [113]. Addressing these challenges requires surface treatments, chemical modifications, or the development of hybrid composites.

From Table 2, it can be seen that incorporating natural fibers can improve the mechanical properties of 3D-printed parts, but the effect depends on factors like fiber content, aspect ratio, and interfacial adhesion. Low fiber loadings often maintain stiffness, while higher loadings can decrease strength. More research is still needed to fully characterize the performance of natural fiber 3D printing of composites under different environmental conditions. However, the current evidence suggests they are a promising sustainable alternative to traditional composites for applications where their mechanical properties are sufficient. To elucidate natural fiber’s potential in 3D printing, this section presents a detailed examination of its compatibility with various 3D printing methods.

#### 4.2.1. Kenaf Fiber

Kenaf fiber, derived from the bast of the kenaf plant, is a promising natural reinforcement material for polymer composites in 3D printing. Known for its high tensile strength and stiffness, kenaf enhances the mechanical properties of thermoplastic matrices such as ABS [109]. When processed into filaments for FDM, kenaf fibers have been shown to improve the overall structural integrity of 3D-printed components. In particular, low to moderate fiber loadings yield optimal results, balancing increased stiffness with retained ductility. Moreover, kenaf fiber’s eco-friendly and biodegradable nature aligns well with the sustainability objectives of modern material science.

Despite its advantages, the integration of kenaf fiber into 3D printing poses significant challenges. These fibers are highly hydrophilic, making them susceptible to moisture absorption [121]. This can lead to swelling, warping, and reduced print quality. Additionally, the natural variability in kenaf fiber length and diameter complicates the process of achieving consistent dispersion within polymer matrices [121]. Another notable challenge is poor adhesion between hydrophilic fibers and hydrophobic polymers, which can lead to weak interfacial bonding and reduced mechanical performance [121]. These issues necessitate surface modifications or coupling agents to enhance compatibility.

Kenaf fiber is particularly well-suited for FDM, where it has demonstrated effective integration with ABS matrices using twin-screw extrusion for filament production [109]. However, its application in resin-based 3D printing methods such as SLA remains limited due to difficulties in dispersing fibers uniformly within liquid resins. Addressing these limitations through advanced processing techniques and material innovations can broaden the applicability of kenaf fiber in additive manufacturing, particularly in fields such as biomedical scaffolds and eco-friendly packaging.

#### 4.2.2. Hemp Fiber

Hemp fiber, extracted from the stalks of the hemp plant, is widely recognized for its versatility and high mechanical strength, making it a valuable reinforcement material for 3D printing. The fiber’s superior tensile modulus and biodegradability contribute to its appeal as a sustainable alternative to synthetic reinforcements. In composite applications, hemp fiber significantly enhances the stiffness and dimensional stability of polymers such as polypropylene (PP) when used in 3D printing processes like FDM [111]. The mechanical properties of hemp-reinforced composites are particularly evident at moderate fiber loadings, where they provide a balance between improved stiffness and retained material flexibility.

However, hemp fibers are inherently hydrophilic, which makes them prone to moisture absorption [122]. This characteristic can cause dimensional instability and reduced mechanical performance during and after the printing process. Additionally, achieving strong interfacial adhesion between hemp fibers and polymer matrices poses a challenge due to their differing surface chemistries [123]. Surface treatments such as alkali or silane treatments are often required to address this issue. Uniform dispersion of fibers within the matrix also remains a challenge, as clumping can lead to inconsistent mechanical properties.

Hemp fibers are particularly compatible with FDM, where their integration into PP-based filaments has been successfully demonstrated [111]. However, their use in resin-based methods such as SLA is constrained by their bulkier structure and low compatibility with liquid resins. Continued research into advanced fiber processing and surface treatments can enhance the viability of hemp fibers in additive manufacturing, paving the way for their use in lightweight structural applications and eco-friendly consumer products.

#### 4.2.3. Harakeke Fiber

Harakeke fiber, obtained from New Zealand flax, is a lightweight and durable natural fiber with significant potential in 3D printing. This fiber is particularly valued for its high stiffness and ability to enhance the mechanical stability of composite materials. When incorporated into polymer matrices such as PP, harakeke fiber improves tensile strength and modulus, making it suitable for applications requiring robust structural properties [111]. Its lightweight nature further contributes to its appeal for use in additive manufacturing, particularly in the production of components where weight reduction is critical.

However, harakeke fiber faces several challenges that hinder its broader application in 3D printing. The fiber’s natural variability in morphology makes achieving uniform dispersion within the polymer matrix difficult. Additionally, harakeke fiber exhibits brittleness at higher loadings, which can reduce the overall ductility of the composite material [124]. Poor interfacial adhesion between the fibers and the polymer matrix, due to their contrasting surface chemistries, further complicates its use. Surface modifications or compatibilizers are often necessary to improve bonding and mechanical performance.

Harakeke fiber has shown strong compatibility with FDM, where its uniform incorporation into PP filaments enhances the performance of printed components [111]. However, its application in other 3D printing techniques, such as SLA or BJT, is limited due to its coarse structure and low compatibility with photopolymers. Addressing these challenges through innovative processing techniques could unlock the full potential of harakeke fiber for diverse additive manufacturing applications.

#### 4.2.4. Wood Dust

Wood dust, a byproduct of woodworking processes, has gained attention as a cost-effective and aesthetic reinforcement material for 3D printing. This fine particulate material is easy to process and integrates well with recycled polymers such as PP [110]. While wood dust does not significantly improve the mechanical properties of composites, it provides a natural and visually appealing finish to 3D-printed components, making it suitable for decorative and low-load applications.

The challenges associated with using wood dust in 3D printing primarily involve achieving uniform dispersion and maintaining printability [125]. The fine particles can clump within the polymer matrix, resulting in inconsistent print quality and weak points in the final product. Furthermore, wood dust’s reinforcement potential is limited com-pared to larger natural fibers, restricting its applicability in structural or high-performance components.

Wood dust has shown compatibility with both FDM and SLA techniques [110]. In FDM, it integrates well with recycled PP filaments, contributing to sustainability efforts. In SLA, the fine particulate nature of wood dust allows for successful incorporation into liquid resins, though its mechanical contributions remain minimal. Future developments in composite formulation and processing could enhance the performance and expand the applications of wood dust in additive manufacturing.

#### 4.2.5. Lignin-Derived Cellulose Nanocrystals

Lignin CNCs represent a cutting-edge material in the field of natural fiber reinforcement for 3D printing. These nanoscale fibers, obtained from the lignocellulosic components of biomass, offer remarkable properties, including a high surface area, exceptional stiffness, and excellent mechanical reinforcement potential. When incorporated into photopolymer resins, lignin CNCs significantly enhance the tensile modulus and overall structural integrity of 3D-printed components [116]. Their nanoscale dimensions enable them to blend seamlessly with resins, providing a uniform reinforcement effect that is difficult to achieve with larger natural fibers.

One of the primary challenges in utilizing lignin CNCs is due to their nanoscale size and stiffness, making them difficult to incorporate into 3D printing processes, particularly those that involve thermoplastic extrusion such as FDM [126]. CNCs can increase the viscosity of the polymer melt, making it more difficult to extrude the material through the printer nozzle, potentially leading to blockages or inconsistent extrusion. Additionally, the high surface area of CNCs increases the likelihood of processing issues like uneven flow and inconsistent printing. These issues are particularly problematic in high-speed printing applications, where precise control over material flow is essential.

Lignin CNCs are particularly well-suited for SLA due to their ability to integrate with liquid photopolymers and their nanoscale size, which minimizes issues with dispersion [116]. SLA-printed composites incorporating lignin CNCs exhibit superior dimensional stability and improved mechanical properties compared to unreinforced resins. Future research could explore hybrid systems where lignin CNCs are combined with other natural fibers to maximize reinforcement while maintaining ease of processing. As advancements in processing techniques and surface modifications continue, lignin CNCs are poised to play an increasingly significant role in additive manufacturing, particularly in high-performance applications requiring nanoscale precision and reinforcement.

## 5. Three-Dimensionally Printed Natural Fiber Composites for Biomedical Application

3D printing, or additive manufacturing, has significantly transformed the healthcare sector by enabling the rapid fabrication of customized medical devices, implants, and scaffolds. Among the various materials employed in 3D printing, natural fiber composites have garnered considerable attention due to their biodegradability, biocompatibility, and mechanical properties, making them especially suitable for healthcare applications. This section aims to explore the role of natural fiber composites in biomedical 3D printing, with an emphasis on their unique advantages, the challenges they face, and their current and future potential in healthcare settings.

### 5.1. Advantages of Using 3D-Printed Natural Fiber Composites in Healthcare

Natural fibers possess complex hierarchical structures that can be utilized in the production of bio-filaments. The primary structure of natural fibers consists of cellulose, hemicellulose, and lignin arranged in different patterns depending on the type of fiber [127]. These structures can be modified and processed to create bio-filaments with specific properties. Cellulose micro-fibrils are the basic building blocks of natural fibers like cotton, hemp, and flax. These micro-fibrils provide strength and stiffness to the fiber and can be extracted and processed to create bio-filaments with high tensile strength and durability. Natural fibers contain various chemical bonds and cross-linkages between polymer chains, which contribute to their mechanical properties. These bonds can be modified or reinforced to create bio-filaments with desired properties such as increased strength or biodegradability. These materials merge the versatility of 3D printing technology with the eco-friendly properties of natural fibers, presenting a compelling solution for sustainable manufacturing across various industries.

Bio-filaments used in 3D printing are composed of a blend of cellulose or nano-cellulose extracted from natural fibers and a biodegradable polymer matrix such as PLA or polyhydroxyalkanoates (PHAs) [128,129]. The biodegradable polymer matrix in bio-filaments ensures that the end products are compostable, minimizing waste and pollution. This feature is crucial in combating the issue of plastic pollution, offering a viable alternative to traditional plastics. Bio-filaments are lightweight yet robust, making them ideal for applications where weight reduction is desirable. Table 3 shows a comparison of the thermal properties of commercialized PLA filament and bio-filaments.

The importance of the melt-mass flow rate (MFR) in 3D printing lies in its ability to predict the flowability of a material during the extrusion process [130]. The MFR is a measure of the rate at which a melted polymer flows through a specific orifice under a specified temperature and pressure. This test is crucial for determining the suitability of a material for 3D printing and ensuring consistent filament properties. In this context, by manufacturing cellulose polymer in the form of a filament, it is possible to replace current thermoplastic materials with bio-filaments [131,132].

**Table 3:** Research on 3D-printed natural fiber composites for biomedical applications.

Matrix	Fiber	Melt-Mass Flow Rate (MFR, g/10 min)	Ref.
Ultimaker PLA	–	6.0	[133]
Spectrum Group PLA	–	6.0	[134]
Ultimaker PP	–	20	[135]
PPprint GmbH PP	–	19.3	[136]
PP	–	22.2	[137]
PP	Nano-fibrillated cellulose (type of fiber was not specified)	20.5–22.0	[137]
PP	–	6.124	[138]
PP	Kenaf	4.560	[138]

In addition, choosing whether to use a low-MFR filament or a high-MFR filament completely depends on the specific printing needs. A low-MFR filament is better for detailed prints and fine features because it tends to flow less easily, allowing for more precise control over the extrusion. It can also result in smoother surfaces and better print quality, especially for intricate designs. Meanwhile, a high-MFR filament flows more easily and quickly through the nozzle, making it suitable for faster print speeds and larger prints. Therefore, it is much easier to print with and less likely to cause clogs in the extruder.

From the comparison shown in Table 3, it is found that most of commercial filaments have a low MFR. Initially, FDM 3D printing was created to make small and intricate pieces of jewelry. Thus, most commercial filaments were made with a low MFR to ensure better print quality for intricate and fine designs. By reviewing the MFR reported in previous research using natural fibers, it can be seen that it is possible to manufacture a filament that is made of natural fibers, since the MFR of cellulose filaments similar to that of commercial filaments. From Table 3, it can also be seen that the MFR of material combined with natural fibers is lower than that of neat polymers, resulting in a better print quality for tissue scaffolds or prosthetic components.

Another advantage of using natural fiber composites in healthcare applications is their excellent biocompatibility and biodegradability [139]. Using extracted cellulose as a 3D printing bio-filament is a promising application that aligns with the growing interest in sustainable materials for tissue engineering and bio-printing. Cellulose, as a natural polymer, is generally biocompatible and non-toxic, making it suitable for biomedical ap-plications. When used as a material for 3D printing, it can provide a supportive matrix for cell growth and tissue formation [140]. The mechanical properties of the printed con-structs are influenced by the composition and structure of the cellulose bio-filament. By modifying factors such as cellulose concentration, cross-linking methods, and incorporation of reinforcing agents, it is possible to tailor the mechanical properties to match those of native tissues. Cellulose bio-filament can be used in multi-material printing approaches, allowing for the fabrication of heterogeneous tissue constructs with varying mechanical and biological properties. Integration with other biomaterials, such as hydrogels or synthetic polymers, can further enhance the functionality of printed tissues. Natural fibers such as kenaf, hemp, and flax possess properties that make them suitable for bio-medical use, particularly in implants or prosthetics [141]. These fibers are well-tolerated by the human body, which is critical when used in medical devices that come into direct contact with tissue. Furthermore, the biodegradability of these fibers enhances their appeal in applications such as temporary implants or tissue scaffolds. These materials gradually degrade over time, reducing the need for secondary surgeries to remove implants, which minimizes patient risk and recovery time.

Another significant advantage lies in the ability to customize medical devices through 3D printing [1]. The precision of 3D printing allows for the production of patient-specific solutions, ensuring a perfect fit for individual anatomical requirements. In the context of bone and tissue engineering, natural fiber-reinforced composites can be tailored to match the mechanical properties of the specific tissue being replaced or repaired. For instance, 3D-printed bone scaffolds made from biodegradable polymers and natural fibers can provide the necessary mechanical strength while supporting cell growth and regeneration. This personalization of medical devices is especially crucial in complex surgeries, where off-the-shelf implants often fail to provide the required fit or functionality.

### 5.2. Current Healthcare Applications of Natural Fiber Composites in 3D Printing

**Figure 9:**
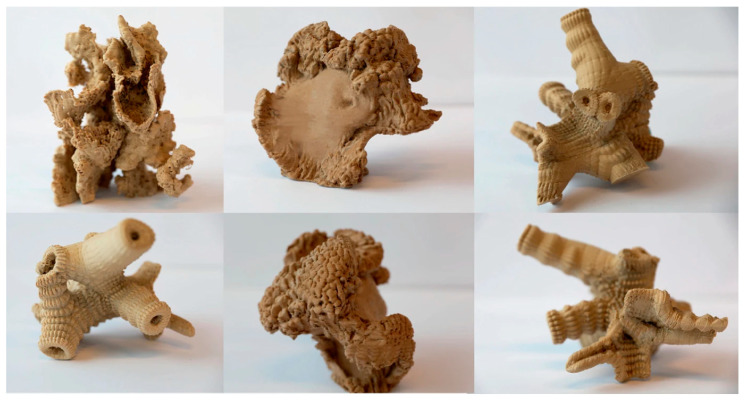
Bio scaffolds for Mycelium growth 3D-printed by Alima et al. [142] using biodegradable polymers infused with cornstarch and natural fibers.

**Figure 10:**
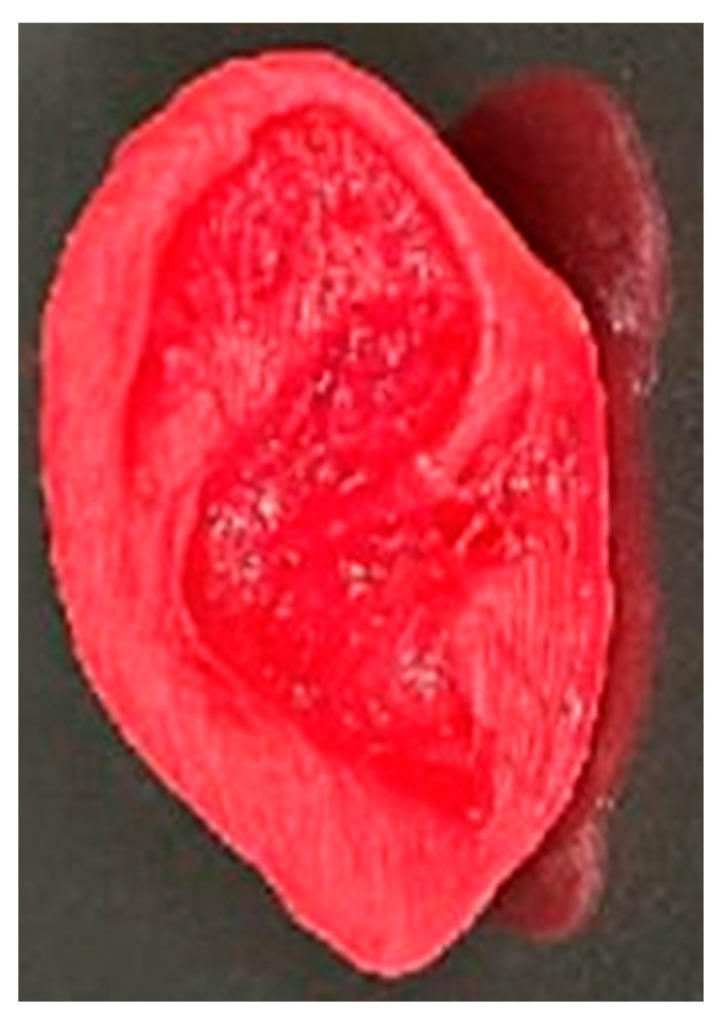
Ear scaffold 3D-printed by Yuan et al. [143] using cellulose.

The use of natural fiber composites in 3D printing is being explored for a wide range of healthcare applications, with some of the most promising uses being in bone and tissue engineering [1,126,129]. Scaffolds 3D-printed from natural fiber-reinforced composites have shown great promise in supporting the regeneration of bone tissue. The mechanical properties of these scaffolds can be engineered to mimic those of natural bone, providing both structural support and a substrate for cell adhesion and growth. Kenaf and hemp fibers, in particular, have been shown to enhance the tensile strength and stiffness of these scaffolds, making them suitable for load-bearing applications in bone regeneration [107]. The biodegradable nature of these composites ensures that the scaffold will gradually degrade as the natural tissue forms, leaving behind only the regenerated bone. Figure 9 and Figure 10 shows some examples of scaffolds that are currently being researched.

**Figure 11:**
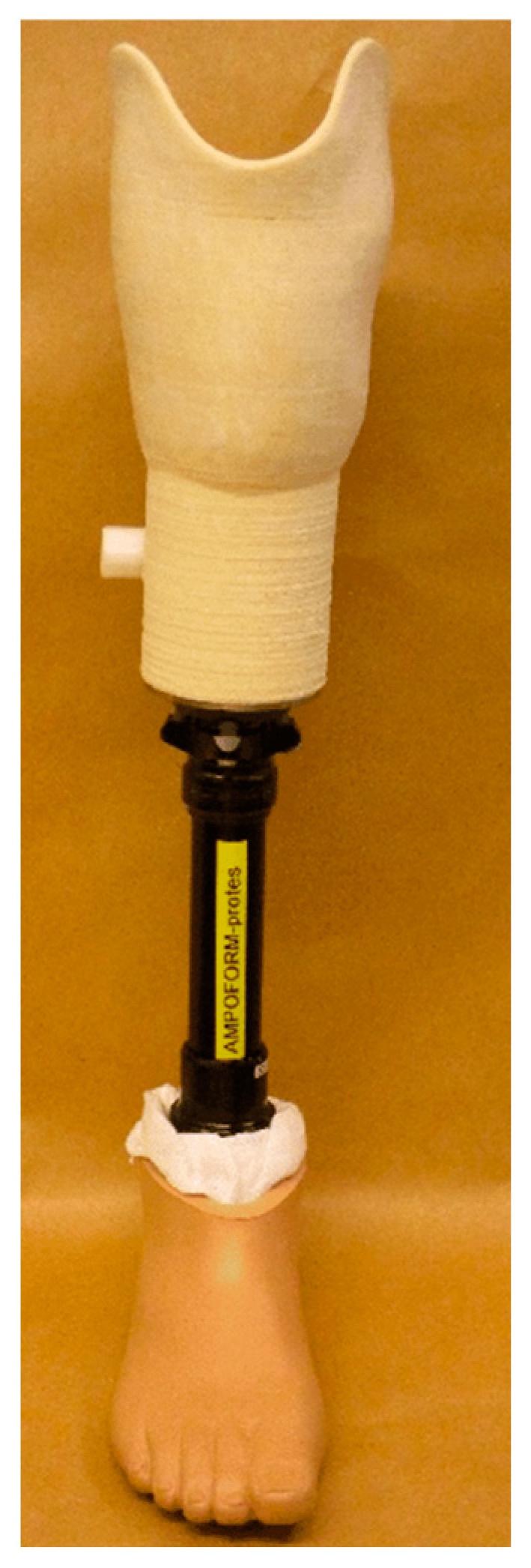
Prosthetics made from 3D-printed natural fiber composites by Stenvall et al. [146].

**Figure 12:**
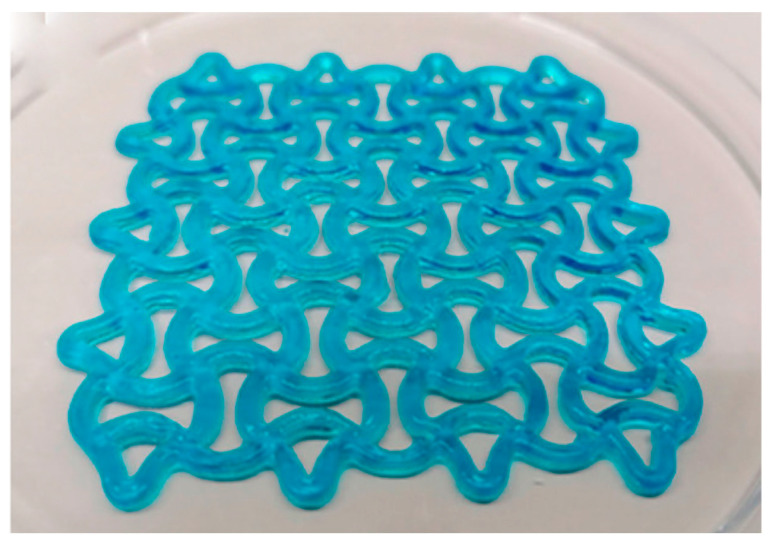
Nanocellulose/chitosan composites 3D-printed by Ajdary et al. [147].

In addition to bone regeneration, 3D-printed natural fiber composites are also being utilized in the development of prosthetics and implants [144]. Natural fibers, due to their lightweight and strong mechanical properties, make an excellent alternative to traditional materials like metals and plastics, which can be heavy and uncomfortable for patients. Three-dimensional printing allows for the production of custom-made prosthetic limbs or joint replacements that perfectly fit the patient’s anatomy, improving both comfort and functionality. These personalized solutions not only offer enhanced mechanical properties but also reduce the need for multiple fittings or revisions typically required with standard, mass-produced devices. Figure 11 shows an example of a 3D-printed prosthetic, and Figure 12 shows a 3D printed nanocellulose/chitosan hydrogel suitable for implants.

Moreover, 3D-printed natural fiber composites are being investigated for their potential use in wound healing and drug delivery systems [145]. In these applications, natural fibers can be incorporated into bioactive materials that provide a scaffold for tissue regeneration while delivering therapeutic agents in a controlled manner. This can improve the healing process by creating an environment that supports cellular activity and the delivery of necessary drugs to the wound site. The biodegradability of these composites ensures that the material breaks down naturally, avoiding the need for surgical removal once its function has been completed.

### 5.3. Challenges and Future Potential in Healthcare Applications of 3D-Printed Natural Fiber Composites

Despite the numerous advantages, the use of natural fiber composites in healthcare applications presents several challenges. One of the primary concerns is material compatibility. While natural fibers are generally biocompatible, achieving optimal performance in composite materials requires careful consideration of their interaction with bio-active polymers or resins [120,148,149]. Natural fibers often require surface treatments or coupling agents to improve adhesion to the polymer matrix [110,150]. Without proper surface treatment, the bond between the fibers and the matrix may be weak, leading to reduced mechanical strength and poor overall performance in the final printed product. This is particularly concerning for biomedical applications, where mechanical properties are crucial for the success of the device.

Another challenge lies in the inherent moisture sensitivity of natural fibers [151,152,153]. These fibers are highly susceptible to absorbing moisture from the environment, which can affect their mechanical properties and stability during the 3D printing process. Moisture absorption can lead to swelling, warping, and inconsistent material behavior, resulting in poor print quality and compromised performance. In applications such as bone scaffolds, where structural integrity is critical, moisture-induced variations can pose a significant risk. To mitigate these issues, it is essential to control the moisture content of the fibers before and during the printing process, which can be both time-consuming and costly.

Regulatory and safety concerns also pose significant challenges for the use of natural fiber composites in health care. Medical devices are subject to rigorous regulatory standards that ensure their safety and effectiveness. While natural fibers are generally considered biocompatible, the composites created with these fibers must undergo extensive testing to meet the required safety standards. This includes tests for cytotoxicity, mechanical performance, and long-term stability within the body. The certification process can be lengthy and expensive, which can delay the adoption of natural fiber composites in healthcare applications.

The future of 3D-printed natural fiber composites in health care is highly promising. Ongoing research is focused on overcoming the challenges related to material compatibility, moisture sensitivity, and regulatory approval. For example, hybrid composites that combine natural fibers with synthetic polymers or nanomaterials may provide enhanced mechanical properties while maintaining the sustainability of natural fibers. These hybrid materials could bridge the gap between the biodegradability of natural fibers and the strength requirements of certain biomedical applications.

In addition to material innovations, the integration of 3D printing with other technologies, such as bioprinting and tissue engineering, holds great potential for the development of advanced medical devices. Natural fiber composites could be used alongside living cells to create more sophisticated tissue scaffolds that not only provide structural support but also actively promote tissue regeneration. This approach could revolutionize regenerative medicine by enabling the creation of custom tissue constructs for transplantation or repair.

Furthermore, the development of functionalized natural fibers for specific healthcare applications, such as antimicrobial properties for wound healing, could significantly expand their use. This would make natural fiber composites even more versatile, enabling them to serve a wider range of medical purposes, from infection-resistant wound dressings to drug delivery systems for chronic conditions.

The use of 3D-printed natural fiber composites in health care represents a transformative approach to creating sustainable, biocompatible, and customizable medical devices. From bone scaffolds to prosthetics and wound healing applications, the potential for these materials to improve patient outcomes is vast. While challenges related to material compatibility, moisture sensitivity, and regulatory approval remain, continued research and technological advancements are poised to address these barriers. As the field progresses, natural fiber composites will play an increasingly important role in shaping the future of health care, offering innovative, eco-friendly solutions for personalized medical care.

## 6. Conclusions

The evolution of AM, more commonly known as 3D printing, has revolutionized numerous industries by offering unprecedented design flexibility, efficiency, and material usage. From its speculative origins in science fiction to the practical applications we see today, AM has traversed a remarkable path. The technology’s ability to build complex geometries layer by layer sets it apart from traditional subtractive manufacturing methods, enabling innovations across the aerospace, healthcare, and automotive industries, in addition to consumer products.

AM’s historical development underscores its transformative potential. The groundwork laid by pioneers such as Johannes F. Gottwald, David E. H. Jones, and Charles Hull has led to the creation of diverse AM technologies, including SLA, SLS, and FDM. These advancements have facilitated the production of intricate components, customized medical devices, and lightweight yet robust aerospace parts, highlighting AM’s versatility and adaptability.

The use of composites in AM represents a significant leap forward. Integrating materials like carbon fiber, fiberglass, and Kevlar into polymer matrices enhances the mechanical properties, resulting in parts that are both lightweight and exceptionally strong. This has broadened the scope of applications, from improving the thermal properties of polymers to developing high-performance components for structural applications. Moreover, ongoing research into new materials such as biodegradable plastics and conductive materials promises to further expand AM’s capabilities.

Despite the advantages, AM faces challenges, including material costs, production speed, scalability, and environmental impact. The high cost of advanced materials and the slower production rates compared to traditional manufacturing methods can hinder widespread adoption. Additionally, scaling AM for mass production requires substantial investment in infrastructure and skilled personnel, which can be a barrier for many industries. Environmental concerns, particularly concerning the energy consumption and carbon emissions associated with AM, also need to be addressed to ensure sustainable growth.

Looking ahead, the future of AM is bright, driven by continuous advancements in technology and materials. The integration of artificial intelligence and machine learning is expected to enhance the efficiency and accuracy of AM processes, paving the way for smarter and more responsive manufacturing systems. Furthermore, the development of new composite materials and hybrid manufacturing techniques will likely open new avenues for innovation.

AM is poised to redefine the manufacturing landscape, offering solutions that are not only efficient and cost-effective but also environmentally sustainable. By harnessing the full potential of 3D printing technologies, industries can achieve greater customization, reduce waste, and accelerate the development of new products. As we move forward, the collaborative efforts of researchers, engineers, and industry leaders will be crucial in overcoming existing challenges and unlocking the full potential of additive manufacturing.

Thus, this review underscores the importance of AM technologies and materials development, particularly the use of 3D-printed composites, in shaping the future of manufacturing and biomedical applications. The possibilities are vast, and the journey of AM has only just begun.

## Figures and Tables

**Table 1:** Previous research on the use of natural fibers for 3D printing.

Printer Type	Polymer	Fiber	Weight Composition (%)	Extruder Model	3D Print Model	Ref.
FDM	ABS	Kenaf	0–10	HTGD-20 twin-screw extruder	FlashForge Creator Pro	[109]
	Recycled PP	Wood dust	0–3	LabTech’s twin screw extruder	–	[110]
	PP	Hemp	0–30	ThermoPrism TSE-16-TC twin screw extruder	Diamond Age	[111]
	PP	Harakeke	0–30	ThermoPrism TSE-16-TC twin screw extruder	Diamond Age	[111]
	PLA	*Hedysarum* *coronarium*	0–20	Polylab single-screw extruder	Next Generation	[112]
	PLA	Agave	0–10	Leistritz Micro 27 GL/GG 32D twin-screw extruder	Wanhao Duplicator 4	[113]
SLA	Poly(ethylene glycol) diacrylate	Abaca CNC	0–1.2	–	Formlabs Form 2	[114]
	Acrylic-based photocurable resin	Wheat straw	5	–	Formlabs Form 1	[115]
	Acrylic-based photocurable resin	Rice straw	5	–	Formlabs Form 1	[116]
	Photoreactive methacrylate (MA) resin	Lignin CNC	0–1	–	Formlabs Form 1+	[116]

**Table 2:** Tensile properties of 3D-printed natural fiber composites.

Technology	Polymer	Fiber	Fiber Loadings (%)	Tensile Strength (MPa)	Tensile Modulus (GPa)	Ref.
FDM	ABS	–	0	23.2	0.33	[109]
	ABS	Kenaf	2.5 to 5	11.48–21.48	0.18–0.33	[109]
	PP	–	0	17	0.9	[111]
	PP	Harakeke	10–30	17–24	1.2–2.3	[111]
	PP	Hemp	10–30	14–16	1.1–2.2	[111]
	PLA	–	0	56	1.26	[112]
	PLA	*Hedysarum* *coronarium*	10–20	62–63	1.84–2.07	[112]
	PLA	–	0	56.7	1.93	[109]
	PLA	Henequen flour	1–5	35.5–60.1	1.43–1.62	[109]
SLA	Poly(ethylene glycol) diacrylate	–	0	0.6	0.026	[114]
	Poly(ethylene glycol) diacrylate	Abaca CNC	0.3–1.2	0.4–1.2	0.026–0.028	[114]

## Appendix A

A timeline of the development of additive manufacturing technology is shown in Figure A1.
